# Knowledge, attitude and practice towards blood donation and associated factors among adults in Debre Markos town, Northwest Ethiopia

**DOI:** 10.1186/s12878-016-0062-8

**Published:** 2016-09-05

**Authors:** Yenework Acham Jemberu, Ahmed Esmael, Kedir Y. Ahmed

**Affiliations:** 1 Community based newborn care officer, JSI/L10K Finote Selam, Ethiopia; 2 School of medicine, College of Medicine and health science, Debre Markos University, PO Box: 269, Debre Markos, Ethiopia; 3 Department of Public Health, College of Medicine and Health Science, Debre Markos University, PO Box: 269, Debre Markos, Ethiopia

**Keywords:** Blood donation, Knowledge, Attitude, Practice

## Abstract

**Background:**

Although the demand for blood supply has progressively increased in developing countries, evidences indicate that there is a major shortage of blood and blood products in these countries, particularly in Ethiopia. Thus, identifying motivational factors affecting blood donation and recruitment of safe and low risk donors is necessary. For this reason, the study aimed at assessing knowledge, attitude, and practice towards blood donation and its associated factors.

**Methods:**

A community based cross-sectional study was conducted in Debre Markos town from February to April, 2015. Multi-stage sampling technique was employed to recruit a total of 845 study participants. Interviewer administered questionnaire was employed as a data collection tool. Binary logistic regression was applied to assess the relationship between explanatory variables and outcome variables.

**Results:**

In this study, 436 (56.5 %) and 403 (52.2 %) were found to be knowledgeable and having favorable attitude, respectively, while the other 124 (16.1 %) reported to have the practice of blood donation. Younger age group, male sex, those who attended formal education and radio listener were significantly associated with the knowledge of blood donation. Attending secondary and above education, having higher income, listening to radio broadcasts, and knowledge of blood donation were found to be the independent predictors of attitude. The practice of blood donation was higher among respondents who were older, attended certificate and above education, knowledgeable, and favorable attitude groups.

**Conclusion:**

The prevalence of knowledge and practice of blood donation is found to be higher compared to similar study conducted in Mekelle, whereas the level of attitude is found to be lower. The finding of this study also justified any possible interventions on the independent predictors. There should be a regularly scheduled awareness creation and voluntary blood donation campaigns organized at the community level to utilize potential blood donors.

## Background

Blood transfusion is a fundamental and a requisite part of any nation’s health care delivery system for a lifesaving interventions. The need for blood and blood products is rising in all parts of the world [1, 2]. Evidence showed that about a quarter million maternal deaths globally and around 15 % of child mortality in Africa were attributed to obstetric bleeding and anemia, respectively [3]. Had there been adequate and safe blood transfusion service such a significant mortality would have been averted. Globally, around 92 million unit blood donations are collected annually from all types of blood donors. The lowest levels of availability are found in low and middle income countries, particularly in Africa [2]. Blood donation rates in Africa is estimated to be 5/1000 populations compared with developed countries which is 47/1000 population in USA [4].

Ethiopia is the second most populous nation in Africa, with an estimated population of 84 million [5]; a country with high MMR of 676/100,000 [6] and high motor vehicle accident (ranks 12^th^ in the world) [7] and with a larger non-immune population for malaria. The Ethiopian Red Cross Society (ERCS) has been the pioneer organization in developing blood banking services in the country. According to ERCS, the country’s blood demand is estimated to be 80,000 to 120,000 units per year. However, only 24,000 units of blood were collected in 2004 (0.3 units/1000 people) and of these 17,000 units (71 % of the total) were collected from Addis Ababa. This figure indicates severe shortage of blood supply affecting the vast majority of the nation’s population (about 96 %) residing outside the capital city [8–10].

Therefore, improving the level of knowledge and hastening the development of positive attitude towards blood donation in the society should be the goal in designing an efficient strategy for sustaining a safe and adequate blood provision throughout the year. The first step to meet this goal should be performing an objective, thorough and comprehensive assessment of knowledge and attitude towards blood donation in the society. A study conducted in the city of Mekelle showed that half of the respondents have a low knowledge about blood donation. Only 10 % have highly supportive attitude and majority (88 %) did not have a history of blood donation [11]. This is lower than that of the study conducted among health science students in Addis Ababa. This study reported that the level of knowledge was 83 % while 68 % of students had favorable attitude, only 23.4 % of them reported history of blood donation [12]. Occupational status, educational level, sex [12, 13], peer pressure, encouraging media and religion [14] were commonly reported independent predictors of KAP of blood donation.

Considering the magnitude and the gravity of the problem, finding few research papers in the area although might sound a little surprising. This makes research in the area profoundly pragmatic. The result of this study will be used in launching an appropriate motivational campaign in the field as there is no community based study conducted in Ethiopia. The aim of this study was thus to assess knowledge, attitude, and practice towards blood donation and its associated factors.

## Methods

### Study design and study setting

Community based cross-sectional study was conducted to assess the knowledge, attitude, and practice towards blood donation and its associated factors among adults in Debre Markos town from February to April 2015.

Debre Markos town is the capital city of East Gojam Zone. It is located 300 Km from the capital of Ethiopia, Addis Ababa and 265 Km from the Amhara Region capital, Bahir Dar. Based on Ethiopian population and housing census 2007 estimation, the total population of the town was estimated to be 101,582, of which 52,827 of them were male [5].

### Source and study population

All adults aged 18 to 65 years residing in Debre Markos town were the source population. Those adults who were selected by multi-stage sampling technique and lived in the study area for at least six months were included. Study participants who had serious illness during the data collection period and unable to hear were excluded from the study.

### Sample size determination

The sample size was determined with a single population proportion formula by taking the prevalence rate of knowledge (49 %) from the Mekelle study [11], which gives the largest sample size. Using 95 % CI and 5 % margin of error, the sample size was found to be 845 after considering 10 % non-response rate and design effect of two.

### Sampling techniques

Multi-stage sampling technique was used to recruit the study participants. At stage one, three Kebeles (the lowest administrative unit in Ethiopia) were selected from the total of seven Kebeles of the town by lottery method. At stage two, systematic random sampling technique was used to select households with proportional allocation to the size of the kebeles. Finally, lottery method was employed to select one study participant for households with more than one eligible individual (aged 18–65 years old).

### Data collection

Data were collected by face-to-face interview by using a structured and pre-tested questionnaire. The questionnaire was first prepared in English and translated to Amharic, then translated back to English by another person to check for its consistency. The questionnaire had five sections comprising; socio-demographic factors (age, sex, marital status, religion, monthly income, educational status and occupation), behavioral factors, knowledge questions (31 questions), attitude questions (12 questions) and practice questions (four questions) of blood donation. Seven diploma nurses and two BSc nurses were recruited as data collectors and supervisors, respectively.

### Data quality control

The quality of data was assured by proper designing of the questionnaire and pre-testing of the questionnaire in one of the Kebeles other than the selected Kebeles. Training was given for the data collectors as well as supervisors by the principal investigator for a day just before and a day after the pretest. During the entire data collection days, all completed questionnaires were reviewed and cross-checked for completeness by the supervisors and all the necessary feedback was given to the data collectors immediately.

### Operational definitions

Based on the mean score, those who score mean and above for knowledge questions were categorized as ‘**knowledgeable**’, otherwise ‘**not knowledgeable**’. And those who score mean and above for attitude question were labeled as having ‘**favorable attitude**’, otherwise ‘**unfavorable attitude**’. Regarding practice, having at least one history of blood donation was used to label them as having practice.

### Data processing and analysis

All the questionnaires were checked visually, coded, and entered into EPI info version 6.0, then transferred to SPSS version 16.0 for analysis. Frequencies, proportions, and measures of central tendency and variation were used for descriptive analysis. Binary logistic regression analysis was used to examine association between the explanatory variables and dependant variables. All variables with *p* value < 0.2 in bivariate analysis were entered in to the final multiple logistic regression model to identify variables independently associated with KAP towards blood donation. Backward stepwise likelihood ratio was used to select the final independent predictors. The significance of Odds Ratio (OR) was declared at *p* value < 0.05.

### Ethical consideration

This Study was reviewed and approved by the Ethical Review Committee of Debre Markos University, College of Medicine and Health Science. Formal permission letter was also obtained from concerned bodies of Debre Markos town. All participants were adequately pre-informed with regard to the aim and the implication of the study and were clearly told as to their full right to refuse or withdraw their verbal consent to participate in the research. Confidentiality of information were kept including omitting personal identifiers such as the name of the respondents.

## Result

### Socio-demographic characteristic of respondents

A total of 845 participants were sampled and 772 (91.4 %) of them were interviewed which yield a non-response rate of 73 (8.6 %). Among respondents, 426 (55.2 %) and 670 (86.8 %) of them were females and had primary school and above educational level, respectively. About 686 (88.9 %) of participants were Orthodox in religion and 389 (50.4) of them were married in marital status. Two hundred fifty two (32.6 %) of them were private workers, while the other 179 (23.2) of them were government employers (Table 1).

**Table 1:** Socio demographic characteristics of respondents for the KAP of blood donation in Debre Markos town, Northwest Ethiopia, 2015 (*n* = 772)

Characteristics	Frequency	Percentage (%)
Age in years		
18–25	292	37.8
26–35	235	30.4
36–45	130	16.8
> 45	115	14.9
Sex		
Male	346	44.8
Female	426	55.2
Religion		
Orthodox	686	88.9
Muslim	68	8.8
Protestant/catholic	18	2.3
Marital status		
Married	389	50.4
Single	335	43.4
Widowed	32	4.1
Divorced	16	2.1
Educational status		
No formal education	102	13.2
Primary (1–8^th^)	66	8.5
Secondary (9–12^th^)	237	30.7
Certificate and above	367	47.5
Occupational status		
Private employer	252	32.6
Government employer	179	23.2
Student	167	21.6
House wife	138	17.9
Daily labor	20	2.6
Other	16	2.1
Monthly Income in Eth. Birr		
< 500	91	11.8
500–1000	165	21.4
1001–1500	91	11.8
> 1500	425	55.1

### Knowledge about blood donation and its associated factors

All of the respondents were heard about blood donation at least once. The majority 601 (71.2 %) of which were heard from television followed by 92 (11.9 %), who were heard from radio. About 415 (53.8 %) and 480 (62.2 %) of them knew that people can donate once in three months, and blood donation can be started at 18 years of age, respectively. Six hundred nine (78.9 %) of respondents knew that blood donation is beneficial for the donor’s health. Majority, 746 (96.6 %) of respondents were reported that voluntary blood donation as the best source of blood supply and less than half (47 %) of them were aware of the risk of transmission of infection by blood transfusion. The risk of transmission of HIV, Hepatitis, Syphilis, and Malaria were mentioned by 46, 11.7, 12.2 and 6.1 % of the respondents, respectively (Table 2). Considering 31 knowledge questions, 436 (56.5 %) of them scored mean and above indicates knowledgeable about blood donation.

**Table 2:** Proportion of respondents who correctly answered knowledge questions among adults in Debre Markos town, Northwest Ethiopia, 2015 (*n* = 772)

Knowledge variables	Frequency	Percentage
Knew place of blood donation	685	88.7
Knew interval of blood donation	415	53.8
Age to start blood donation	480	62.2
Minimum criteria for blood donation		
Age 18–65 years	286	37.0
weight above 45 kg	231	29.9
Basic good health	540	69.9
Blood donation is useful for donors health	609	78.9
Importance of blood donation		
Free health checkup	107	13.9
Healthy feeling	167	21.6
Feeling of happiness	563	72.9
Reduction of heart attack	55	7.1
Burns calories and control body weight	34	4.4
Lower risk of cancer	25	3.2
Best source of blood donation	746	96.6
There is such thing as artificial blood	521	67.5
Possible health risks of blood donation		
Anemia	251	32.5
Cross infection	59	7.6
Fainting	220	28.5
Sudden death	32	4.1
HIV positive person can donate blood	666	86.3
Knew any infections transmitted by transfusion	363	47.0
Infection can transmit during blood transfusion		
HIV/AIDS	355	46.0
Hepatitis	90	11.7
Syphilis	94	12.2
Malaria	47	6.1
Who are not eligible for blood donation		
Infectious disease patients	606	78.5
Pregnant and lactating women	369	47.8
History of major surgery in past year	190	24.6
History of blood transfusion	119	15.4
Intra venous drug users	53	6.9

The study showed that males (AOR = 1.89 95 % CI: 1.36, 2.61) were more knowledgeable than females. Those who were at the age range of 18–25 (AOR = 2.42 95 % CI: 1.33, 4.40) and 26–35 (AOR = 2.06 95 % CI: 1.13, 3.73) years were more knowledgeable compared to reference age groups of 45 years and above. Being at primary school level (AOR = 2.93 95 % CI: 1.39, 6.17), at secondary school level (AOR = 3.33 95 % CI: 1.68, 6.60) and Certificate and above educational level (AOR = 4.39 95 % CI: 2.29, 8.39) were significantly associated with knowledge of blood donation compared to those who have no formal education. The odds of being knowledgeable among radio broadcast listeners (AOR = 0.37 95 % CI = 0.19, 0.75) was significantly lower than internet and/or newspaper users (Table 3).

**Table 3:** Factors associated with knowledge towards blood donation among adults in Debre Markos Town, Northwest Ethiopia (*n* = 772)

Variables	Knowledgeable		COR 95 % CI	AOR 95 % CI
	Yes	No		
Sex				
Male	229	281	2.07 (1.55, 2.78)	1.89 (1.36, 2.61)**
Female	207	367	1	1
Age (years)				
18–25	191	101	5.14 (3.18, 8.26)	2.42 (1.33, 4.40)*
26–35	150	85	4.78 (2.92,7.80)	2.06 (1.13, 3.73)*
36–45	64	66	2.63 (1.54, 4.49)	1.38 (0.75, 2.54)
> 45	31	84	1	1
Educational status				
Non formal education	18	84	1	1
Primary school (1–8^th^)	30	36	3.89 (1.93, 7.85)	2.93 (1.39, 6.17)*
Secondary (9–12^th^)	147	90	7.62 (4.30, 13.5)	3.33 (1.68, 6.60)**
Certificate & above	241	126	8.93 (5.14, 15.5)	4.39 (2.29, 8.39)**
Source of information				
Television	355	246	0.64 (0.38, 1.07)	1.03 (0.60, 1.76)
Radio	29	67	0.19 (0.10, 0.37)	0.37 (0.19, 0.75)*
Newspaper/Internet	52	23	1	1

*COR*, crude odds ratio, *AOR* adjusted odds ratio

**p-value < = 0.001, *p-value < 0.05

Bold data indicates significant association

### Attitude of blood donation and its associated factors

Among respondents, 598 (77.5 %) of them showed willingness to donate blood in the future. Of these, 571 (95.4 %) of them wanted to donate blood as voluntary donor and, the rest 25 (4.3 %) and 2 (0.3 %) of them as replacement and paid donors, respectively (Table 5). Regarding the composite measure of attitude, about 403 (52.2 %) of the respondents had favorable attitude towards blood donation.

The likelihood of favorable attitude towards blood donation was higher among those who attended secondary school (AOR = 5.15 95 % CI: 2.81, 9.43) and certificate and above educational level (AOR = 4.85 95 % CI: 2.70, 8.72) compared to adults without formal education. The odds of favorable attitude was higher (AOR = 1.87 95 % CI: 1.13, 3.09) in 1500 Ethiopian Birr (ETB) and above monthly income groups. Radio listener had lower chance (AOR = 0.43 95 % CI: 0.21, 0.87) of having favorable attitude compared to newspaper and/or internet user. Being knowledgeable towards blood donation had increased (AOR = 1.40 95 % CI: 1.01, 1.94) odds of favorable attitude towards blood donation compared to its counterparts (Table 4).

**Table 4:** Factors associated with attitude towards blood donation among adults in Debre Markos Town, Northwest Ethiopia (*n* = 772)

Variables	Attitude		COR 95 % CI	AOR 95 % CI
	Favorable	Unfavorable		
Educational status				
Non formal education	18	84	1	1
Primary school (1–8^th^)	20	46	2.03 (0.98, 4.22)	1.65 (0.77, 3.54)
Secondary school (9–12^th^)	140	97	6.74 (3.81, 11.92)	5.15 (2.81, 9.43)**
Certificate & above	225	142	7.39 (4.26, 12.83)	4.85 (2.70, 8.72)**
Average Monthly Income				
< 500 Birr	38	53	1	1
500–1000 Birr	66	99	0.93 (0.55, 1.56)	0.87 (0.5, 1.53)
1000–1500 Birr	28	63	0.62 (0.34, 1.14)	0.52 (0.27, 0.99)
> 1500 Birr	271	154	2.45 (1.55, 3.89)	1.87 (1.13, 3.09)*
Use of media equipment				
Television	329	272	0.68 (0.41,1.12)	0.85 (0.51, 1.43)
Radio	48	27	0.21 (0.11, 0.40	0.43 (0.21, 0.87)*
Newspaper/internet	26	70	1	1
Knowledge				
Knowledgeable	171	265	2.23 (1.66, 2.97)	1.40 (1.01, 1.94)*
Not knowledgeable	198	138	1	1

*COR* crude odds ratio, *AOR* adjusted odds ratio

**p-value < = 0.001, *p-value < 0.05

Bold data indicates significant association

### Practice of blood donation and its associated factors

The practice of blood donation was found to be 124 (16.1 %) and of these, 79 (63.7 %) and 36 (29 %) of them were donated once, and twice, respectively. However, only 6 (4.8 %) of them were regular donors. Among those who ever donated blood, majority 79 (63.7 %) of them were voluntary donors and, the remaining 45 (36.3 %) were donated to a friend or relative in need of blood. The reasons for non-donation were inability to think of it 219 (33.8 %), lack of opportunity 212 (32.7 %), lack of time 72 (11.1 %), fear of pain 62 (9.6 %), fear of knowing their serostatus 9 (1.4 %) and non-remuneration 4 (0.6 %) (Table 5).

**Table 5:** The intention and practice of blood donation among adults in Debre Markos Town, Northwest Ethiopia, 2015 (*n* = 772)

Variable	Frequency	Percentage
History of blood donation		
Yes	124	16.1
No	648	83.9
Frequency of donation		
Once	79	63.7
Twice	36	29
Three times	9	7.3
Type of blood donation		
Voluntary	79	63.7
Friends/relative	45	36.3
Regular donors		
Yes	6	4.8
No	118	95.2
Willingness in the future		
Ye	598	77.5
No	174	22.5
Type of donation intended		
Voluntary	571	95.5
Replacement	25	4.2
Paid	2	0.3
Reasons of not blood donation		
Didn’t think of it	219	33.8
Lack of opportunity	212	32.7
Lack of time	72	11.1
Fear of pain	62	9.6
Fear knowing my status	9	1.4
No remuneration	4	0.6
Others	70	10.8

The likelihood of blood donation was higher among 18–25 years (AOR = 0.42 95 % CI: 0.19, 0.92) and 26–35 years (AOR = 0.26 95 % CI: 0.11, 0.63) of age groups compared to the reference group of 45 and above year olds. Participants whose level of education was certificate and above were more likely (AOR = 7.40 95 % CI: 3.04, 8.86) to donate blood compared to those who have no formal education. The odds of blood donation was found to be higher (AOR = 3.17 95 % CI: 1.90, 5.28) among knowledgeable respondents compared to their counterparts. The odds of blood donation was 5.19 times (95 % CI: 5.19 (3.04, 8.85)) higher among those with favorable attitude than unfavorable attitude (Table 6).

**Table 6:** Factors associated with practice towards blood donation among adults in Debre Markos Town, Northwest Ethiopia (*n* = 772)

Variables	Practice		COR with 95 % CI	AOR with 95 % CI
	Yes	No		
Age (years)				
18–25	39	253	1.11 (0.58, 2.14)	0.27 (0.11, 0.65)*
26–35	39	196	1.44 (0.75, 2.77)	0.26 (0.11, 0.63)*
36–45	32	98	2.36 (1.19, 4.68)	0.79 (0.33, 1.94)
> 45	14	101	1	1
Educational status				
Non formal education	4	98	1	1
Primary school	3	63	1.17 (0.25, 5.38)	1.01 (0.2, 5.09)
Secondary school	21	216	2.38 (0.80, 7.12)	2.08 (0.58, 7.42)
Certificate and above	96	271	8.68 (3.11, 24.23)	7.40 (3.04, 8.86)**
Knowledge				
Knowledgeable	100	336	3.87 (2.42, 6.20)	3.17 (1.90, 5.28)**
Not knowledgeable	24	312	1	1
Attitude				
Favorable	104	299	6.07 (3.67, 10.04)	5.19 (3.04, 8.85)**
Unfavorable	20	349	1	1

*COR* crude odds ratio, *AOR* adjusted odds ratio

**p-value < = 0.001, *p-value < 0.05

Bold data indicates significant association

## Discussion

The overall level of knowledge towards blood donation was found to be 56.5 % which is higher than community based study conducted in the city of Mekelle (49 %) [11]. However, it is lower than another study conducted among health science students in Addis Ababa (83 %) [12]. The difference in socio-economic status with residents of Mekelle and in educational status with the health science students might explain the discrepancy with the above findings. About 53.8 % of participants knew that people can donate every 3 months, this is higher than other studies conducted in Benin, (21.5 %), Chennai (51.2 %) and Mekelle (43.6 %) [11, 15, 16]. The majority of them reported voluntary blood donation as the best source of blood donation. This is consistent with finding from Chennai [16], however it is higher than studies conducted among health workers (72.2 %), and physicians in Benin (80.7 %) [15–17]. Less than half (47 %) of adults knew the risk of transmission of disease through blood transfusion which is lower than that of the study conducted in Benin (95.7 %) [15] and, higher than that of the study conducted in India [18].

Age, sex, educational status and source of information were found to be independent predictors of knowledge of blood donation. Male study participants were more knowledgeable towards blood donation which is in line with findings from Karachi and North India [19, 20]. In Ethiopian context, males are more accessible to information and spent most of their time out of their home than females. Having at least primary education was significantly and positively associated with the knowledge of blood donation, which is supported by a study conducted in Sikim, India [13]. This could be because more educated people might be in a better position to access the media and availability of awareness creation at primary and secondary school and higher educational institutions. Participants who used radio as a source of information were less knowledgeable than those who used newspaper and/or internet. This might be partly explained by the limitations of radio programs on addressing the intended goals. On the other hand the low level of knowledge found in a radio listeners might be due to their limited access, techno illiteracy and their limited educational preparedness to understand, analyze and absorb the content of the message.

The composite measure of attitude based on mean score indicates that 52.2 % of the respondents had favorable attitudes towards blood donation. This is better than a study conducted in Karachi (42 %), however it is lower than similar studies conducted in India (87.3 %), Mekelle (61 %) and Addis Ababa (68 %) [11, 12, 19, 21]. More than three-fourth of respondents had intention to donate blood in the future which is lower than other studies conducted in India (90 %) and Addis Ababa (100 %) [12, 18]. Studies conducted in India and Addis Ababa carried out among health science students showed that knowledge of blood donation was higher as a result of their profession.

The present study found that educational status, average monthly income, source of information, and knowledge were significantly associated with favorable attitude towards blood donation. Participants who had higher monthly income were more likely to have favorable attitude than lower income groups. This might be because those who have higher income may access better information sources. Radio listener had lower chance of having favorable attitude compared to newspaper reader and internet users. This might be partly explained by the limitations of radio programs on addressing the intended goals. On the other hand, the low level of knowledge found in a radio listeners might explain the unfavorable attitude of them as knowledge is an independent predictor of attitude.

In addition, about 16.1 % of respondents reported at least one history of blood donation which is lower than similar studies conducted in Benin [15], Northern Nigeria [22], South India [21], and in Addis Ababa [12]. However, it is higher than other studies carried out in India (13.9 %) [18] and Mekelle (12 %) [11]. The difference in the practice of blood donation could be due to variation in the setup of study settings since some of the studies were conducted among health professionals and some others were at school level. Nearly two-third of blood donation were practiced as voluntary blood donation. This is supported by the study conducted in South India (64.1 %) [21]. About 4.8 % of blood donors were regular donors, which is very low compared to similar study conducted in Benin (13.9 %) and facility based study from Addis Ababa (42.2 %) [12, 15]. The major reasons reported from those who didn’t practice blood donation were inability to think of it (33.8 %), lack of opportunity to donate blood (32.7 %), and lack of time (11.1 %). Global researchers also concluded that people are not donating blood because nobody approached them for donation, lack of information, unfit to donate, a need to donate for a friend or relative in future, fear of needle and knowing their viral status, the donated blood may be sold, non-remuneration, ignorance and their religion [12, 15, 17, 18, 21, 23].

Age, educational status, knowledge and attitude were significantly associated with the practice of blood donation. Older age groups were positively associated with the practice of blood donation. This is similar with findings from Karachi and Iran [21, 24]. This might be attributed to increased personal experience from donating blood. Having certificate and above educational level was associated with increased practice of blood donation which is comparable with the study carried out in Addis Ababa [12]. This might support the fact that education can positively influence the knowledge and attitudes of blood donation. In addition, being knowledgeable and having favorable attitude were found to be independent predictors of blood donation. Knowledge of blood donation is a pre-requisite in obtaining access to and providing voluntary blood donation timely and effectively. It is also an important tool in avoiding fear and building positive attitude of blood donors. This is supported by a similar study conducted in Mekelle [11].

The fact that this study was conducted at the community level could be mentioned as the strength of the study. The nature of cross-sectional study which is not possible to establish cause-effect relationship between the explanatory variables and outcome variables, and the possibility of social desirability bias were among the limitations of this study.

## Conclusion

The present study showed that the prevalence of knowledge and practice of blood donation is found to be higher compared to similar study conducted in Mekelle, whereas the level of attitude is found to be lower. Younger age group, male sex, who attended formal education and radio listener were significantly associated with knowledge of blood donation. Attending secondary and above education, having higher income, listening to radio broadcasts, and knowledge of blood donation were found to be the independent predictors of attitude. The practice of blood donation was higher among respondents who were older, attended certificate and above education, were knowledgeable, and had favorable attitude. The finding of this study also justified any possible interventions on the independent predictors. There should be a regularly scheduled awareness creation and voluntary blood donation campaigns organized at the community level to utilize potential donors who lack time and opportunity to donate blood.

## Abbreviations

AOR: Adjusted odds ratio
COR: Crude odds ratio
ERCS: Ethiopian Red Cross Society
KAP: Knowledge attitude and practice
